# Effects of Intraosseous Tibial vs. Intravenous Vasopressin in a Hypovolemic Cardiac Arrest Model

**DOI:** 10.5811/westjem.2015.12.28825

**Published:** 2016-03-02

**Authors:** Justin Fulkerson, Robert Lowe, Tristan Anderson, Heather Moore, William Craig, Don Johnson

**Affiliations:** U.S. Army Graduate Program in Anesthesia, Fort Sam Houston, Texas

## Abstract

**Introduction:**

This study compared the effects of vasopressin via tibial intraosseous (IO) and intravenous (IV) routes on maximum plasma concentration (Cmax), the time to maximum concentration (Tmax), return of spontaneous circulation (ROSC), and time to ROSC in a hypovolemic cardiac arrest model.

**Methods:**

This study was a randomized prospective, between-subjects experimental design. A computer program randomly assigned 28 Yorkshire swine to one of four groups: IV (n=7), IO tibia (n=7), cardiopulmonary resuscitation (CPR) + defibrillation (n=7), and a control group that received just CPR (n=7). Ventricular fibrillation was induced, and subjects remained in arrest for two minutes. CPR was initiated and 40 units of vasopressin were administered via IO or IV routes. Blood samples were collected at 0.5, 1, 1.5, 2, 2.5, 3, and 4 minutes. CPR and defibrillation were initiated for 20 minutes or until ROSC was achieved. We measured vasopressin concentrations using high-performance liquid chromatography.

**Results:**

There was no significant difference between the IO and IV groups relative to achieving ROSC (p=1.0) but a significant difference between the IV compared to the CPR+ defibrillation group (p=0.031) and IV compared to the CPR-only group (p=0.001). There was a significant difference between the IO group compared to the CPR+ defibrillation group (p=0.031) and IO compared to the CPR-only group (p=0.001). There was no significant difference between the CPR + defibrillation group and the CPR group (p=0.127). There was no significant difference in Cmax between the IO and IV groups (p=0.079). The mean ± standard deviation of Cmax of the IO group was 58,709±25, 463pg/mL compared to the IV group, which was 106,198±62, 135pg/mL. There was no significant difference in mean Tmax between the groups (p=0.084). There were no significant differences in odds of ROSC between the tibial IO and IV groups.

**Conclusion:**

Prompt access to the vascular system using the IO route can circumvent the interruption in treatment observed with attempting conventional IV access. The IO route is an effective modality for the treatment of hypovolemic cardiac arrest and may be considered first line for rapid vascular access.

## INTRODUCTION

Each year in the United States more than 326,000 out-of-hospital cardiac arrests occur.^1^ In fact, cardiac arrest remains the leading cause of morbidity and mortality with more than 900 occurrences daily in the U.S.^1^,^2^,^4^ Hemorrhage with subsequent cardiac arrest is the leading cause of death on the military battlefield as well as in civilian trauma.^2^ When a patient is in cardiac arrest, it is essential to establish rapid and reliable vascular access. Research has shown that survival rate depends on a rapid sequence of therapeutic interventions including vascular access.^3^–^7^ The chances of survival is worsened for every minute that drugs are delayed.^5^,^8^,^9^ In a cardiac arrest scenario particularly from hypovolemic shock, the patient’s veins have collapsed preventing vascular access. This makes the procedure not only difficult but time consuming, which could delay administration of life-saving drugs. In emergent conditions, trained providers take significantly longer and more attempts to establish vascular access via conventional peripheral intravenous (IV) insertion than the intraosseous (IO) approach.^6^ In current military operations, in pre-hospital emergencies, and mass casualties, there are many additional environmental and tactical obstacles to overcome while attempting to establish vascular access.

The IO route provides access to a rapidly obtained, non-collapsible, venous plexus.^8^ The American Heart Association (AHA), the European Resuscitation Council (ERC), the American College of Emergency Physicians (ACEP), the American Academy of Pediatrics (AAP), the American College of Surgeons (ACS), the U.S. National Association for Emergency Medical Service Physicians (NAEMSP), and the U.S. Army Committee on Tactical Combat Casualty Care (TCCC) recommend the use of IO access if IV access is not readily available.^3^,^10^–^16^ The recommendation is based on limited evidence that the route is effective for drug administration during a cardiac arrest. Two variables relative to IO drug administration have the potential to alter the pharmacokinetics and subsequent return of spontaneous circulation (ROSC): vascular distribution to the bone marrow and flow to the bone. Bone marrow changes structure and composition with age. At birth, bone contains primarily red marrow, which is highly vascularized. After five years of age, the red marrow is replaced by yellow marrow, which is significantly less vascular. By adulthood, red marrow is found primarily in the sternum, proximal femur, humerus and skull. IO site selection may be important given the variability of blood flow to these different types of marrow.^17^ Also, when a patient is in hypovolemic shock, endogenous catecholamines and subsequent vasoconstriction to the bone may lead to less flow from the bone. We speculated that the when a patient is in cardiac arrest, tibial IO compared to IV administration of vasopressin would result in lower concentrations, lower maximum plasma concentrations (Cmax), and the time it takes to reach maximum concentration in plasma (Tmax). We also speculated that hypovolemia would alter drug distribution and affect the concentration, Cmax, and Tmax reducing the chances of ROSC. Furthermore, we reasoned the time to achieve ROSC would be more for IO compared to IV administration.

No research studies have evaluated the pharmacokinetics of vasopressin-administered IO compared to IV in a hypovolemic model. Furthermore, no study has addressed ROSC using tibial IO vasopressin in the hypovolemic model in a cardiac arrest model. The purposes of this study were to compare ROSC, time to ROSC, serum concentration of vasopressin, Cmax, Tmax, and odds of survival relative to administration by IV and IO tibia routes compared with control groups that received cardiopulmonary resuscitation (CPR) and defibrillation and one that received just CPR.

Specifically, the following research questions guided the study:

1. Are there statistically significant differences in ROSC and time to reach ROSC between the groups?
2. Are there statistically significant differences in Cmax and Tmax of serum vasopressin when administered via tibial IO and IV routes?
3. Are there statistically significant differences in mean concentration of vasopressin over four minutes between the tibial IO and IV routes?
4. What are the comparative odds of su0rvival by group?

## METHODS

### Design and Sample

The study was a prospective, between-subjects, experimental design. The Institutional Animal Care and Use Committee (IACUC) approved the research protocol, and the animals received care in compliance with the Animal Welfare Act. Twenty-eight Yorkshire swine were randomly assigned by a computer generated random number program to one of four groups: IV + defibrillation (n=7), IO tibia + defibrillation (n=7), CPR + defibrillation (n=7), and a control group that received just CPR (n=7). Two additional swine were added in the each of the IV and IO groups for model development. These two swine meet the criteria for inclusion in the study, and no changes were necessary in the protocol. Therefore, the pigs were included in both groups to make a total of eight in both the tibial IO and IV groups. However, one pig in the IO group was ill and was deleted from the study making a total of seven in that group.

Swine were selected because the cardiovascular system and bone are comparable to humans. In addition, their blood volume is the same as humans: 70ml per kg of body weight.^18^,^19^ To avoid any variability in subjects, we purchased the swine from the same vendor and acquired pigs that were approximately the same size. Male swine were used to avoid any potential hormonal effects. Subjects weighing between 60 to 80kg were used as this range represents the average weight of an adult, male human.^20^ They were observed for three days to ensure they were in a good state of health. All subjects received no food after midnight the evening before the study but were allowed fluids as desired until the experiment

### Procedures

Each subject received pre-emptive analgesia with Telazol (4–8mg/kg). They were then sedated, anesthetized, intubated, and placed on mechanical ventilation. A standard Narkomed^®^ anesthesia machine (Dräger, Telford, PA) was used to deliver isoflurane at a maintenance dose (0.5–2%) and ventilation at 8–10mL/kg at 10–14 breaths per minute. A peripheral IV was started on all subjects using an 18- or 20-gauge catheter in the auricular vein. The peripheral auricular vein was chosen because it is most comparable to the antecubital vein in humans.^21^ Hemodynamics were evaluated continuously that included the following: electrocardiogram, arterial blood pressure via a left carotid artery catheter, mean arterial pressure, oxygen saturation, end-tidal carbon dioxide, and temperature. A forced-air warming blanket was used to sustain rectal temperature greater than 36 degrees Celsius. A Vigileo™ (Edwards Lifesciences, Irvine, CA) was used to obtain cardiac output and stroke volume measurements via the arterial line. The femoral artery was cannulated for the collection of blood samples and for the achievement of controlled hemorrhage.

For swine in the tibial IO group, we inserted an EZ-IO^®^ device (Vidacare, San Antonio, TX) in the proximal, medial tibia. Placement was confirmed by aspiration of blood and easy irrigation with 10mL of 0.9% normal saline (NS). Patency was maintained by administering Lactated Ringer’s solution with a pressure bag at 300mmHg. Subjects were allowed to stabilize for 15 minutes; we then exsanguinated 31% of their blood volume from the femoral artery catheter into a canister. This represented a Class III hemorrhage. Hemorrhage was accomplished by allowing blood to drain by gravity over approximately 15 minutes. To ensure the amount exsanguinated was correct, the investigators used a scale that was accurate and precise within 0.5%.

In response to hypovolemic shock in accordance with Tactical Combat Casualty Care guidelines, we administered 500mL of 5% albumin to all subjects over 10 minutes.^22^ Five minutes after the administration of albumin, the investigators placed the pigs in cardiac arrest, defined as a nonperfusing arrhythmia. Specifically, after we visualized the heart on transthoracic ultrasound, one spinal needle was placed superior and one placed inferior to the heart. The needles were attached to alligator clamps. The clamps were attached to three 9-volt batteries that were connected in a series to deliver an electrical current, thereby inducing nonperfusing ventricular fibrillation. We were able to establish ventricular fibrillation usually within 10 seconds. In some cases, we had to reposition the needles resulting in 100% being placed into ventricular fibrillation.

The pigs were allowed to remain in arrest for two minutes. Then CPR was initiated by use of the Michigan Automated Thumper™ (Michigan Instruments, Grand Rapids, MI) to automatically compress the sternum to a predetermined depth of 1½ inches at a rate of 100 compressions per minute as per guidelines of the AHA.^3^ The device ensured consistency and reproducibility of quality chest compressions across all subjects. CPR continued for two minutes with ventilations delivered at 10 breaths per minute.

Vasopressin was then administered at a dose of 40 units to the IV and IO subjects. The drug was rapidly injected IV or IO push followed by 20mL of NS flush. Blood samples (10mL) were collected from the femoral artery catheter every 30 seconds for three minutes and again at four minutes after vasopressin injection. Before each sample was collected, 10mL of blood was collected and discarded to avoid sample contamination. The catheter was then flushed with 10mL of NS to maintain patency. A baseline sample was not necessary because the drug contains arginine while endogenous vasopressin in swine exclusively contains the lysine isoform.

After the samples were collected, we defibrillated the swine starting at 200 joules. If a nonperfusing rhythm persisted, the pigs were defibrillated with 360 joules. CPR continued on all subjects, and pigs that remained in ventricular fibrillation were defibrillated at 360 joules every two minutes. CPR was continued for 20 minutes or until ROSC. The investigators defined ROSC as the presence of a sustained perfusing heart rhythm, palpable femerol pulse, and systolic blood pressure (SBP) of ≥60mmHg. Defibrillation was not initiated earlier because any pigs that achieved ROSC before all samples were collected would confound the analyses of drug pharmacokinetics. Subjects that achieved ROSC were monitored for an additional 30 minutes. For all groups, arterial blood gases (ABG) were obtained every five minutes to determine the effectiveness of the treatment modalities. The same procedures for the CPR + defibrillation group were used as above, but vasopressin was not administered and no samples were collected. For the CPR only group, these subjects did not receive vasopressin or defibrillation. To determine mean concentration and Cmax, the investigators used a liquid chromatography with mass spectrometry (HPLC-MS/MS). The HPLC method is considered to be the gold standard in pharmacokinetic research.^23^ One trained person, who was blinded to group assignment, performed all of the HPLC analyses, specifically the mean concentration and Cmax. For the purposes of this study, Cmax was defined as the peak or highest concentration of serum vasopressin. The mean concentration of was defined as the arithmetic average of each time a sample was collected.

### Statistical Analyses

The investigators used data from similar, previous studies and calculated a large effect size of 0.6.^24^–^26^ Using an α of 0.05, an effect size of 0.6, and a power of 0.80, the investigators determined a sample size of 28 (n=7 per group) was needed. We performed power analysis using G*Power 3.1 for Windows (Heinrich Heine University, Dusseldorf, Germany).

IBM^®^ SPSS^®^ Statistics v.17 software (Chicago, IL) was used for data analysis. We calculated means, standard deviations, and standard error of the mean for the IO and IV groups. A chi-square was used to determine if there were differences in ROSC between groups. We used a multivariate analyses of variance (ANOVA) to determine if there were significant differences between the groups relative to the pretest data, the time to ROSC, Cmax, and Tmax. A repeated measures ANOVA (RANOVA) was used to determine if there were statistical differences between groups (measured at 30 second intervals) regarding the mean concentrations over four minutes. We calculated and compared the odds of ROSC by each group.

## RESULTS

There was no significant difference in pretest data by group (weight, amount of hemorrhage, cardiac output, stroke volume, systolic blood pressure, diastolic blood pressure, mean arterial blood pressure, temperature, and pulse) indicating the groups were equivalent on those variables (p>0.05). There was no significant difference between the IO and IV groups relative to achieving ROSC (p=1.0) but a significant difference between the IV compared to the CPR + defibrillation group (p=0.031) and IV compared to the CPR-only group (p=0.001). There was a significant difference between the IO group compared to the CPR + defibrillation group (p=0.031) and IO compared to the CPR-only group (p=0.001). There was no significant difference between the CPR + defibrillation group and the CPR group (p=0.127). The number of subjects achieving ROSC (See Table 1), and the odds of survival were compared by group (See Table 2).

**Figure 1:**
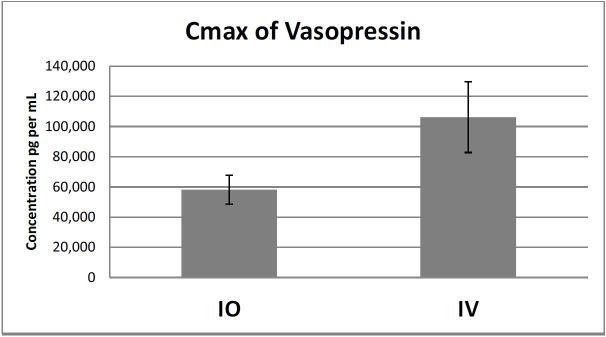
Comparison of Cmax by group. *IO*, intraosseous; *IV*, intravenous

There was no significant difference in Cmax between the IO and IV groups (p=0.079). The mean ± standard deviation of Cmax of the IO group was 58,709 ± 25,46 pg/mL compared to the IV group, which was 106,198 ± 62,135pg/mL (Figure 1).

**Figure 2:**
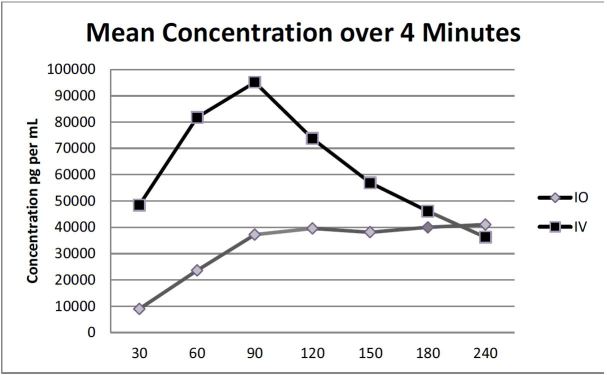
Mean concentration of vasopressin in seconds over four minutes. *IO*, intraosseous; *IV*, intravenous

There was no significant difference in mean Tmax between the groups (p=0.084). The times are in seconds ± standard deviations for the IO and IV groups respectively and were as follows: 158 ± 78.8 and 86 ± 70. There was also no significant difference in time to ROSC by group. (See Table 3 for a summary.) The overall mean concentration of vasopressin over four minutes between the IO and IV groups was not significant (p=0.365). However, a pairwise comparison indicated a significant difference at 60 seconds (p=0.021) between IO and IV groups (mean ± standard error 23,595 ± 14,856pg/mL vs. 76,787 ± 1,896pg/mL respectively (Figure 2).

## DISCUSSION

The purposes of this study were to compare ROSC, time to ROSC, serum concentration of vasopressin, Cmax, Tmax, and odds of survival relative to administration by IV and IO tibia routes compared with control groups that received CPR + defibrillation and one that received just CPR in a hypovolemic swine model. The results are consistent with the findings of Von Hoff, et al. who found there were no statistically significant differences between the Cmax or Tmax after IO (iliac crest) and IV administration of morphine sulfate in humans. However, these authors caution that there may be differences between IO and IV resuscitation drugs and other IO sites. 27 The results of our study support the findings of Johnson, et al. who compared the humerus IO and IV administration of epinephrine and found no statistically significant difference in Cmax, Tmax, ROSC, or time to ROSC. However, Johnson et al. found that at 30 seconds, the mean concentration of epinephrine was higher in the humerus IO group compared to the IV group.^28^ Conversely, in the current study, we found that the mean concentration of vasopressin was consistently higher in the IV compared to the IO group. Our results are consistent with the findings of Burgert et al., Hoskins et al., and Wenzel et al. who found that IV was higher than tibial IO administration of drugs.^5^,^25^,^29^ Specifically, Wenzel et al. found vasopressin administration in a swine model of pediatric cardiac arrest resulted in a comparable rate of ROSC compared to IV vasopressin.^29^ Voelckel et al. found that blood flow decreased significantly during hemorrhagic shock, which they speculated would impair absorption of drugs administered by the IO route in a pediatric model. 30 We did find that the concentration and Cmax was lower the tibial IO compared to IV but these findings may be because of the yellow marrow and distance from the heart. The current study adds to the body of knowledge in that we investigated not only the pharmacokinetics but also ROSC and time to ROSC in a hypovolemic, adult cardiac arrest model. We consistently found the mean concentration and the Cmax to be lower, and the Tmax to be longer in the IO group compared to the IV group. However, these findings did not affect ROSC. Specifically, we found there were no significant differences in the IO and IV groups relative to ROSC or time to ROSC.

## LIMITATIONS

The primary limitation was that experimenters were not blinded to group assignment, but the protocol was followed with the same rigor regardless of group assignment. Another limitation was that the results of the study may not be not generalizable to humans; however, the bone and cardiovascular system are comparable to humans.^18^,^19^ The study used a small sample size and the reader should be cautioned that only two in the CPR + defibrillation group had ROSC. In addition, there was no statistically significant difference in Cmax between the IV and IO groups, but the IO group had 55% of the concentration compared to the IV group. This suggests that the study was underpowered relative to this variable. With a larger sample size, we probably would have found a statistically significant difference. However, further studies are warranted to use a larger sample size. In addition other IO sites need to be used to determine and compare pharmacokinetics and the effectiveness of those sites. We also acknowledge that the study did not have strict adherence to advanced cardiovascular life support (ACLS) guideline relative to defibrillation. We did not want the swine to have ROSC before all of the samples were collected. CPR and a beating heart may have yielded different findings relative to the kinetics and not because of routes of administration. If ACLS guidelines had been followed, we reasoned that ROSC in both the IV and IO groups may have been shorter, but the current study strongly suggest that both routes of administration are effective.

## CONCLUSION

This study illustrates that prompt access to the vascular system using IO insertion can circumvent the interruption in treatment observed with attempting conventional IV access. Time is of the essence when treating cardiac arrest. The time to acquire IV access would certainly take longer even with a skilled provider than the 10 seconds it took us to insert the IO device. Studies show that the time to establish IV access in a variety of settings ranges from 2–49 minutes.^31^–^33^ Administration of vasopressin by IO and IV achieved excellent survival rates indicating both are effective methods of access. Based upon these findings, the IO route might be considered the first choice for rapid vascular access with vasopressin administration for a hypovolemic patient in cardiac arrest.

## Figures and Tables

**Table 1:** Subjects achieving return of spontaneous circulation (ROSC).

Group	Number achieving ROSC	Number not achieving ROSC
IV (N=8)	7(87.5%)	1(12.5%)
CPR + defibrillation (N=7)	2(28.6%)	5(71.4%)
IO (N=7)	6(85.7%)	1(14.3%)
CPR only (N=7)	0(0%)	7(100%)

*IV*, intravenous; *CPR*, cardiopulmonary resuscitation; *IO*, intraosseous

**Table 2:** Comparison of odds of ROSC by group.

Group comparison	Odds of ROSC	Confidence interval	P value
IV vs. CPR + defibrillation	17.5	1.2232 to 250.3694	0.03*
IV vs. CPR only	75	2.6133 to 2152.4792	0.01*
IV vs IO	1.6	0.0593 to 22.9378	0.10
IO vs. CPR + defibrillation	15	1.0306 to 218.3109	0.04*
IO vs. CPR only	65	2.2384 to 1887.4682	0.01*
CPR + defibrillation vs. CPR only	33	1.3059 to 833.9222	0.03*

*ROSC*, return of spontaeous circulation; *IV*, intravenous; *CPR*, cardiopulmonary resuscitation; *IO*, intraosseous

* Significant at the 0.05 level.

**Table 3:** Comparison of time to ROSC by group.

Comparisons	Mean ± standard deviations in seconds	P value
IV vs. IO	559±231	0.619
IO vs. CPR + defibrillation	493±226	0.069
CPR + defibrillation vs. IV	863±181	0.127

*ROSC*, return of spontaeous circulation; *IV*, intravenous; *IO*, intraosseous; *CPR*, cardiopulmonary resuscitation
